# Patterns of Circulating Fibroblast Growth Factor 21 in Subjects with and without Type 2 Diabetes

**DOI:** 10.1371/journal.pone.0142207

**Published:** 2015-11-05

**Authors:** Jingyi Lu, Haoyong Yu, Yifei Mo, Xiaojing Ma, Yaping Hao, Wei Lu, Huating Li, Yuqian Bao, Jian Zhou, Weiping Jia

**Affiliations:** 1 Department of Endocrinology and Metabolism, Shanghai Jiao Tong University Affiliated Sixth People’s Hospital, Shanghai Diabetes Institute, Shanghai Key Laboratory of Diabetes Mellitus, Shanghai Clinical Center for Diabetes, Shanghai, PR China; 2 Department of Endocrinology and Metabolism, Kashgar Prefecture Second People's Hospital, Kashi, Xinjiang, PR China; University of Louisville School of Medicine, UNITED STATES

## Abstract

**Background:**

Fibroblast growth factor 21 (FGF21) exerts wide-range effects on carbohydrate and lipid metabolism. However, its perturbation in type 2 diabetes mellitus (T2DM) remains elusive. Besides, previous human studies in T2DM simply investigated fasting or stimulated levels of FGF21. The current study sought to evaluate the temporal changes of circulating FGF21 in subjects with and without T2DM.

**Methods:**

Ten patients with T2DM and 16 normal controls (NC) were recruited. Participants were categorized as obese (BMI≥25 kg/m^2^) or lean (BMI<25 kg/m^2^). Blood samples were drawn every 30 min within 7 hours (8 a.m.-3 p.m.). Serum FGF21, blood glucose, insulin, free fatty acids (FFAs) and adiponectin were measured in all subjects.

**Results:**

The peak levels of FGF21 were observed in the fasting state (8 a.m.) both in T2DM and NC groups (267.35 ±158.72 ng/L vs. 178.93±121.37 ng/L, P = 0.096). FGF21 AUC did not differ significantly between the two groups (T2DM: 949.4±471.47 ng/L; NC: 883.13±561.40 ng/L, P = 0.770). Obese subjects had higher FGF21 levels than lean ones in patients either with or without T2DM. The pattern of FFAs closely resembled that of FGF21. Correlation analysis showed that temporal levels of FGF21 were significantly related to FFAs (r = 0.749, *P* = 0.002),but not blood glucose, insulin or adiponectin (all *P*> 0.05).

**Conclusions:**

These findings suggest that the pattern of circulating FGF21 does not differ significantly between T2DM and NC,although T2DM patients showed a trend toward higher fasting FGF21 than healthy subjects. The pattern of circulating FFAs is significantly associated with that of FGF21.

## Introduction

Fibroblast growth factor 21 (FGF21) belongs to a subfamily of the FGFs that function as the endocrine,which exerts wide-range effects on carbohydrate and lipid metabolism[1].For instance, FGF21-overexpressing mice were protected from diet-induced obesity, and therapeutic administration of FGF21 improved plasma glucose and triglyceride concentrations both in ob/ob and db/db mice[2]. Similarly, the treatment of diabetic monkeys with FGF21 corrected insulin resistance and improved β-cell function[3].These data indicates that FGF21 may serve as promising targets for the treatment of type 2 diabetes mellitus (T2DM). In humans, several studies reported that the circulating FGF21 levels in patients with T2DM were significantly higher than that in controls without diabetes, presumably due to FGF21 resistance[4–6].

Serum concentrations of hormones engaged in metabolic regulation often display unique diurnal patterns, and aberrant circadian rhythms are closely associated with metabolic abnormalities[7, 8]. In terms of FGF21, our previous study[9] demonstrated that circulating FGF21 levels exhibit a characteristic diurnal rhythm in non-diabetic individuals, manifesting as a major nocturnal rise occurring between midnight and early morning. Most human studies investigating serum FGF21 in the state of diabetes simply used fasting or stimulated FGF21 levels. However, less is known about the temporal changes of FGF21 in T2DM. The elucidation of the temporal changes of FGF21 may provide new insights into the pathophysiology and etiology of T2DM. Therefore, the current study was conducted to measure FGF21 and other metabolic parameters within 7 hours in subjects with and without T2DM.

## Methods

### Study subjects

Ten patients with T2DM were recruited for the study. Inclusion criteria were: 1) established T2DM diagnosed according to the 1999 WHO definition[10]; 2) age of 18 to 70 years; 3) body mass index (BMI) ≥ 18.5kg/m^2^. Exclusion criteria included: 1) history of cardiovascular, severe liver or kidney disease 2) history of psychiatric illness; 3) current treatment with insulin and/or thiazolidinediones. Sixteen healthy subjects were included as the control group (without diabetes, without history of cardiovascular, severe liver or chronic kidney disease and without medication). The clinical characteristics of the T2DM patients are described in Table 1. The clinical characteristics of 16 healthy subjects have been described previously [9]. Blood samples were drawn from an indwelling venous catheter in the forearm every 30 min within a period of 7 hours. The first sample was drawn at 8 a.m. after an overnight fast. The subjects were kept staying at the metabolic ward and received the same standardized meals of natural type: breakfast at 8 a.m. and lunch at 11 a.m. Anthropometric parameters were measured before breakfast. The BMI of each subject was calculated as weight/height^2^(kg/m^2^). A BMI of ≥25 kg/m^2^ was defined as obese according to the Asian-Pacific obesity criteria proposed by the WHO Western Pacific Regional Office.Blood pressure was measured on the right arm after a 20-minute rest.

**Table 1:** Characteristics of T2DM patients.

N	10
Male/female	10/0
Age (years)	55.1±9.9
BMI (Kg/m^2^)	26.6±4.8
HbA1c (%)	8.8±2.6
Systolic BP (mmHg)	134.5±17.6
Diastolic BP (mmHg)	88.2±12.9
Total cholesterol (mmol/L)	5.10±1.03
Triglyceride (mmol/L)	2.96±2.69
HDL-C (mmol/L)	1.09±0.27
LDL–C (mmol/L)	2.98±0.98

Continuous variables are presented as means ± SD; categorical variables are presented as numbers; BP, blood pressure; HDL-C, high density lipoprotein cholesterol; LDL-C, low density lipoprotein cholesterol.

Written consents were obtained from all volunteers. The study protocol was approved by the ethics committee of Shanghai Sixth People’s Hospital affiliated to Shanghai Jiao Tong University. All procedures were conducted in accordance with the Declaration of Helsinki.

### Biochemical measures

Serum FGF21 levels were measured with in-house chemiluminescence immunoassays (CLIA) as previously described[9], and the intra-assay and inter-assay coefficient of variation (CV) was 4.2–5.6% and 5.8–7.3%, respectively. Glucose, triglyceride, total cholesterol (TC), low density lipoprotein-cholesterol (LDL-C), and high density lipoprotein-cholesterol (HDL-C) concentrations were determined enzymatically on a Hitachi 7600 chemical analyzer (Hitachi). Serum concentrations of insulin and free fatty acids (FFAs) were measured with RIA (Linco Research) and enzymatic colorimetric assay (Roche Diagnostics), respectively.

### Statistical analysis

Comparisons of circulating FGF21 levels between groups were conducted with Mann-Whitney U test. Spearman’s correlation coefficient analyses were performed to determine the associations of FGF21 with specific parameters. Area under the curve (AUC) was calculated using the trapezoidal rule.SPSS version 11.0 software (SPSS Inc., Chicago, IL, USA) was used for statistical analysis. A two-sided *P* value of less than 0.05 was considered statistically significant.

Because comparative data about the pattern of circulating FGF21 have not yet been published, the estimate of sample size was based on the feasibility of conducting this study, and a convenience sample size of 26 (T2DM: n = 10; NC: n = 16) was finally adopted.

## Results

**Fig 1:**
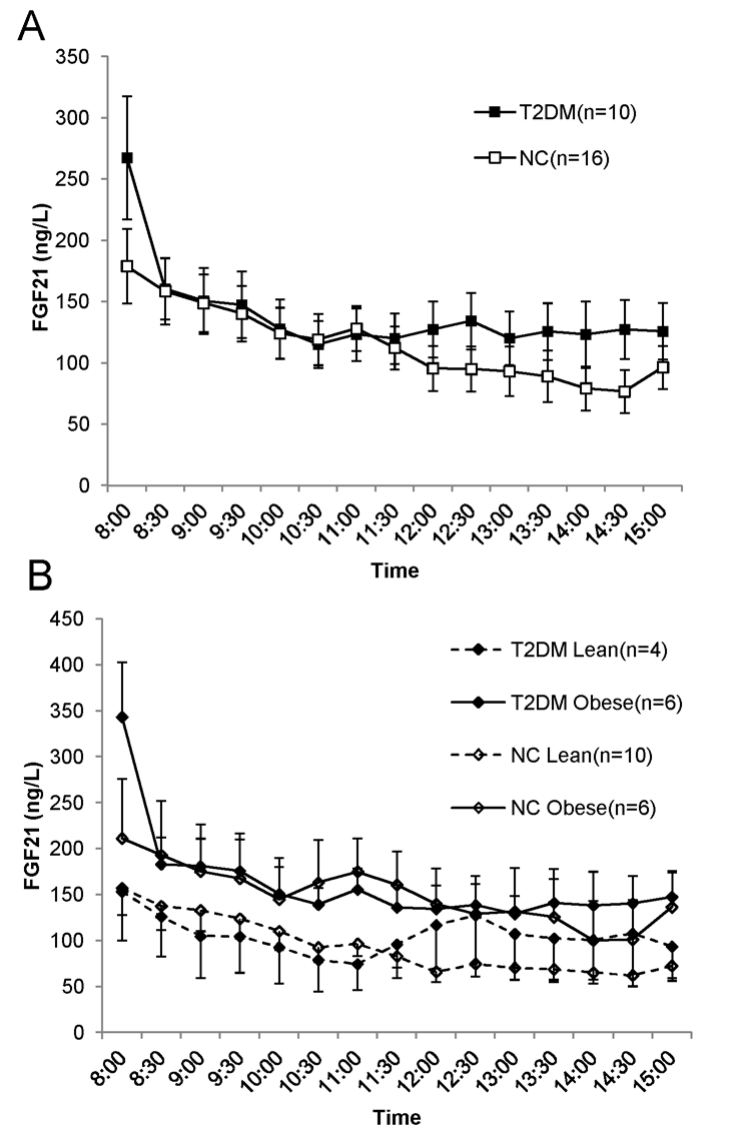
The profiles of FGF21 over 7 hr in subjects with and without T2DM. (A), the comparison of profiles of FGF21 between T2DM and NC. Black squares represent T2DM patients; white squares represent healthy controls. (B), the comparison of profiles of FGF21 between subgroups stratified by BMI in subjects with and without T2DM. Black diamonds represent T2DM patients; white diamonds represent normal controls; solid lines represent obese subjects; dashed lines represent lean subjects. Blood samples were drawn every 30 min from participants who received 2 standardized meals (breakfast: 8 am; lunch 11 am). Data are shown as mean ± SEM.

As illustrated in Fig 1A, the peak levels of FGF21 were observed in the fasting state (8a.m.) both in T2DM and NC groups (267.35 ±158.72 ng/L vs. 178.93±121.37 ng/L, *P* = 0.096). During the next 3 hours, the FGF21 of patients with T2DM seemed to overlap with that of healthy subjects. the average concentrations of FGF21 during 7 hours in T2DM and NC were 130.57±37.51 ng/L and 115.7±30.54 ng/L, respectively (*P* = 0.054).The AUCs of FGF21 did not differ significantly between the 2 groups (T2DM: 949.4±471.47 ng/L; NC: 883.13±561.40 ng/L, *P* = 0.77) (Table 2).

**Table 2:** Characteristics of FGF21 in subjects with and without T2DM.

	T2DM	NC	*P*-value
7-hr Mean (ng/L)	130.57±37.51	115.7±30.54	0.054
Fasting-8AM (ng/L)	267.35±158.72	178.93±121.37	0.096
Peak (ng/L)	267.35±158.72	178.93±121.37	0.096
Nadir (ng/L)	115.06±60.41	76.68±70.53	0.065
AUC (ng/L*7hr)	949.4±471.47	883.13±561.40	0.770

Data are presented as means ± SD; AUC, area under the curve.

We further stratified the study participants according to BMI. Fig 1B depicts that both in T2DM and NC groups, obese subjects had higher FGF21 levels than their lean counterparts, although the difference were not statistically significant (data not shown). Besides, there were no evident differences in FGF21 between T2DM and NC in subgroups of BMI.

**Fig 2:**
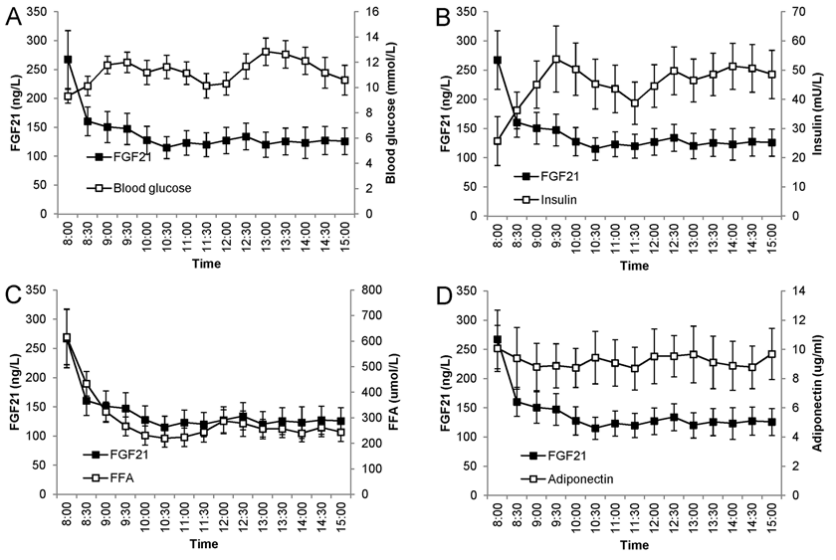
Association of temporal levels of FGF21 with glucose (A), insulin (B), FFAs (C), and adiponectin (D) in T2DM patients. Blood samples were drawn every 30 min from participants who received 2 standardized meals (breakfast: 8 am; lunch 11 am). Data are shown as mean ± SEM.

**Fig 3:**
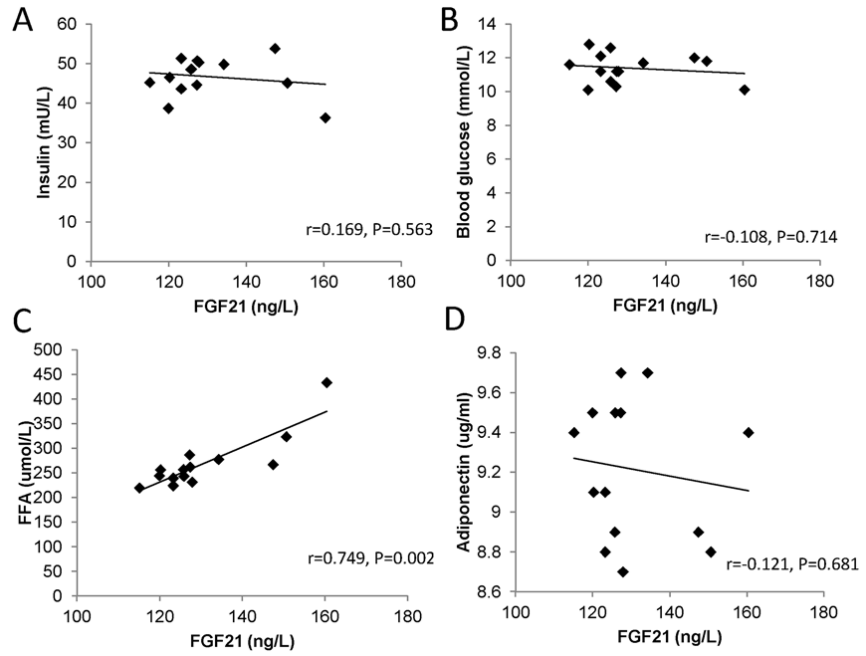
Correlation of the changes in temporal levels of FGF21 with blood glucose (A), insulin (B), FFAs (C), and adiponectin (D) in T2DM patients. Correlation coefficients and P values are from Spearman’s correlation test.

To evaluate the associations of FGF21 levels with other metabolic parameters, blood glucose, insulin,FFAs, and adiponectin were measured in patients with T2DM. Although the patterns of glucose and insulin seemed to be opposite to that of FGF21 (Fig 2A and 2B), the profiles of FFAs showed remarkable similarity to the FGF21 profiles (Fig 2C). In addition, we did not find a clear association between the patterns of adiponectin and FGF21 (Fig 2D). Correlation analysis showed that the FGF21 levels were significantly related to FFAs (r = 0.749, *P* = 0.002), but not blood glucose, insulin or adiponectin (all *P*>0.05) (Fig 3).

## Discussion

As far as we know, this is the first report of changes in the temporal circulating levels of FGF21 in patients with T2DM. We demonstrated that the average concentrations of FGF21 during 7 hours did not differ significantly between subjects with and without T2DM,although patients with T2DM had higher fasting FGF21 levels than did normal controls. Concentrations of FGF21 were significantly correlated with FFAs, but not blood glucose, insulin or adiponectin.

Several previous studies have examined the perturbations of FGF21 levels in patients with T2DM [4, 5, 11]. For example, Chavez et al.[4] measured the plasma FGF21 in 41 subjects with varying degrees of obesity and glucose tolerance. They found FGF21 levels in patients with T2DM were significantly higher than those with impaired glucose tolerance (IGT) or normal glucose tolerance (NGT). Similarly, Chen et al.[5] reported that baseline FGF21 levels increased progressively with worsening dysglycemia from NGT, through prediabetes, to T2DM, suggesting the baseline FGF21 levels were predictive of the future development of diabetes. In this work, although T2DM patients seemed to have higher fasting FGF21 than NC, the difference was not statistically significant, and the AUCs of FGF21 levels over time did not differ significantly between the 2 groups. We speculate that the small sample size could at least partially account for this discrepancy. However, it cannot be excluded that T2DM and NC may differ in the diurnal rhythm but not overall circulating levels of FGF21. For instance, we observed that the obvious decrease in FGF21 between 8:00am and 8:30am was steeper in T2DM. Intriguingly, it was reported that that FGF21 levels in healthy individuals peaks at around 5:00am and declines to a nadir in the afternoon [9]. Therefore, it is possible that T2DM patients may lag behind healthy subjects in the peak time of FGF21 (e.g., 6:00am in T2DM), leading to the difference in fasting circulating FGF21 between the 2 groups, which may be an interesting issue to address in the future.

We found the profiles of FFAs closely resemble those of FGF21, and the concentrations of FGF21 significantly correlated with those of FFAs. In consistence with our findings, Mai et al.[12] found an increased FGF21 expression induced by incubation of HepG2 cells with FFAs, through the activation of peroxisome proliferator–activated receptor α (PPARα), which is an important regulator of FGF21[6, 13]. Moreover, in the human study, lipid infusion in vivo induced an increase in circulating FGF21 levels.

The association between the temporal circulating levels of FGF21 and blood glucose was not statistically significant in this study. Interestingly, there was evidence that circulating levels of FGF21 in patients with newly diagnosed T2DM is similar to that in normal controls[14]. More importantly, FGF21 levels in patients with classic type 1 diabetes mellitus (T1DM)and latent autoimmune diabetes in adults (LADA) are actually lower than that in age- and sex-matched healthy controls[14, 15]. Taken together, these data suggest that hyperglycemia may not play a major role in the modulation of FGF21.

In the current study, the temporal association of insulin with FGF21 did not reach statistical significance. To date, conflicting data has been reported with respect to the insulin-FGF21 relationship. In a community-based study comprising 232 participants, serum FGF21 was found to be positively correlated with fasting insulin [16]. By contrast, patients with T1DM receiving insulin therapy were reported to have lower fasting FGF21 levels as compared with T2DM patients, although insulin concentrations were comparable between the two diabetic groups [15]. Of note, most previous studies examined the relationship of FGF21 with insulin in the fasting state, while little is known about their temporal association. Zibar et al. [17] recently demonstrated in patients with T1DM that, circulating FGF21 did not change significantly after the injection of ultrashort-acting insulin (before standardized meal). Their findings, together with the non-significant result of our work, led us to postulate that circulating FGF21 may be associated with insulin resistance but not insulin itself.

Recently, a mechanistic link between FGF21 and adiponectin was identified. Holland et al.[18] reported that FGF21 administration acutely enhanced adiponectin secretion in mice.It reduced blood glucose levels and improved insulin sensitivity only when functional adiponectin was present. Likewise, Lin et al.[19] demonstrated that the favorable effects of FGF21 on insulin signaling in liver and skeletal muscle were abrogated in adiponectin knockout mice, while FGF21-mediated activation of ERK1/ERK2 in adipose tissues remained intact. With respect to the relationship of FGF21 with adiponectin, previous studies have provided inconsistent data. In a 28-day clinical trial, LY2405319 (an FGF21 Analog) elevated plasma adiponectin in obese subjects with T2DM[20]. However, in vitro treatment of human preadipocytes with FGF21 for the entire differentiation period suppressed the expression and release of adiponectin [21]. Our present study failed to find the significant association between FGF21 and adiponectin. One possible explanation for this finding could be that FGF21 at physiological levels has no impact on the expression and/or secretion of adiponectin.

Serum FGF21 levels in this study were within 150–250 ng/L, consistent with previous reports [5, 22]. It is noteworthy that, although animal studies have convincingly demonstrated beneficial effects of FGF21 on lipid and glucose metabolism, the resulting circulating FGF21 levels after exogenous administration of recombinant FGF21 in these studies were much higher than those in physiological conditions.For example, in the study by Coskun et al. [23], continuous infusion of recombinant FGF21 for 2 weeks attained steady-state circulating levels of 7.4–42.7ng/ml. Therefore, it is tempting to hypothesize that FGF21 may exert its favorable effects in an autocrine or paracrine manner. In accordance with this notion, it has been reported that FGF21 is an inducible, autocrine factor in adipose tissue that functions in a feed-forward loop to regulate the activity of peroxisome proliferator-activated receptor γ (PPARγ) [24], a major transcriptional regulator of adipogenesis. Further studies are needed to clarify the role of FGF21 in energy metabolism as an autocrine or paracrine factor.

In summary, the pattern of serum FGF21 concentrations in the present study did not differ significantly between patients with T2DM and normal controls, although fasting FGF21 in T2DM patients had the trend to be higher than healthy subjects.The temporal levels of FGF21 were significantly associated with FFAs, but not blood glucose, insulin or adiponectin. Future studies measuring the 24-hour profile of FGF21 in larger sample of patients with T2DM are warranted to confirm our observations.

## Supporting Information

S1 Dataset Circulating levels of FGF21 over 7 hours in patients with and without T2DM. (XLSX)
